# Establishment of head and neck squamous cell carcinoma mouse models for cetuximab resistance and sensitivity

**DOI:** 10.20517/cdr.2023.62

**Published:** 2023-10-17

**Authors:** Hannah Zaryouh, Ines De Pauw, Hasan Baysal, Jöran Melis, Valentin Van den Bossche, Christophe Hermans, Ho Wa Lau, Hilde Lambrechts, Céline Merlin, Cyril Corbet, Marc Peeters, Jan Baptist Vermorken, Jorrit De Waele, Filip Lardon, An Wouters

**Affiliations:** ^1^Center for Oncological Research (CORE), Integrated Personalized & Precision Oncology Network (IPPON), University of Antwerp, Campus Drie Eiken, Antwerp 2610, Belgium.; ^2^Pole of Pharmacology and Therapeutics (FATH), Institut de Recherche Expérimentale et Clinique (IREC), UCLouvain, Brussels B-1200, Belgium.; ^3^Institut Roi Albert II, Department of Medical Oncology, Cliniques Universitaires Saint-Luc, Brussels B-1200, Belgium.; ^4^Department of Medical Oncology, Antwerp University Hospital, Edegem 2650, Belgium.; ^#^The authors contributed equally.

**Keywords:** HNSCC, cetuximab resistance, xenograft mouse model, immunodeficient mice

## Abstract

**Aim:** Acquired resistance to the targeted agent cetuximab poses a significant challenge in finding effective anti-cancer treatments for head and neck squamous cell carcinoma (HNSCC). To accurately study novel combination treatments, suitable preclinical mouse models for cetuximab resistance are key yet currently limited. This study aimed to optimize an acquired cetuximab-resistant mouse model, with preservation of the innate immunity, ensuring intact antibody-dependent cellular cytotoxicity (ADCC) functionality.

**Methods:** Cetuximab-sensitive and acquired-resistant HNSCC cell lines, generated *in vitro*, were subcutaneously engrafted in Rag2 knock-out (KO), BALB/c Nude and CB17 Scid mice with/without Matrigel or Geltrex. Once tumor growth was established, mice were intraperitoneally injected twice a week with cetuximab for a maximum of 3 weeks. In addition, immunohistochemistry was used to evaluate the tumor and its microenvironment.

**Results:** Despite several adjustments in cell number, cell lines and the addition of Matrigel, Rag2 KO and BALB/C Nude mice proved to be unsuitable for xenografting our HNSCC cell lines. Durable tumor growth of resistant SC263-R cells could be induced in CB17 Scid mice. However, these cells had lost their resistance phenotype *in vivo*. Immunohistochemistry revealed a high infiltration of macrophages in cetuximab-treated SC263-R tumors. FaDu-S and FaDu-R cells successfully engrafted into CB17 Scid mice and maintained their sensitivity/resistance to cetuximab.

**Conclusion:** We have established *in vivo* HNSCC mouse models with intact ADCC functionality for cetuximab resistance and sensitivity using the FaDu-R and FaDu-S cell lines, respectively. These models serve as valuable tools for investigating cetuximab resistance mechanisms and exploring novel drug combination strategies.

## INTRODUCTION

Head and neck squamous cell carcinoma (HNSCC) is a type of cancer originating in the mucous membranes of the oral cavity, pharynx, and larynx. With over 800,000 patients being diagnosed every year^[1]^, HNSCC remains a challenging disease to treat. Over the years, novel treatment options have emerged targeting the tumor in a much more specific way, thereby reducing unwanted side effects associated with more conventional therapies, such as chemo- and radiotherapy. These novel immuno- and targeted therapies include the anti-programmed cell death-1 (PD-1) monoclonal antibody (mAb) pembrolizumab and the anti-epidermal growth factor receptor (EGFR) mAb cetuximab, which are now, respectively, first- and second-line therapy for the treatment of recurrent/metastatic (R/M) HNSCC^[2-4]^. In addition, the PD-1 antibody nivolumab was already approved in 2016 for the treatment of patients with R/M HNSCC who have progressed on or after platinum-based chemotherapy^[5]^. Although these therapies have proven their worth in terms of prolonging survival and tolerability, the development of therapeutic resistance, leading to a lack of durable efficacy, is a major roadblock in the search for effective treatment options in HNSCC. Finding a way to overcome this resistance might contribute to the much-needed progress in the field. Based on our own previous, extensive research on cetuximab resistance^[6-9]^ as well as preclinical and clinical studies reported in literature^[10-12]^, inhibition of the phosphatidylinositol 3-kinase (PI3K)/Akt pathway might be a promising therapeutic strategy to increase response to EGFR blockade with cetuximab. Recently, we demonstrated that the addition of the Akt inhibitor MK2206 to cetuximab treatment resulted in synergistic effects in both cetuximab-sensitive and acquired cetuximab-resistant HNSCC cell lines^[8]^. However, *in vivo* validation of this combination strategy to overcome cetuximab resistance is yet to be investigated. Although cell lines grown in simple two-dimensional (2D) culture systems are useful and informative for initial screening of anti-cancer drugs, *in vivo* evaluation is essential for several reasons. Firstly, *in vivo* studies can provide information on the pharmacokinetics and toxicity of a drug, which cannot be accurately predicted by *in vitro* tests. Secondly, *in vivo* studies can reveal the efficacy of a drug in reducing tumor growth and metastasis, which is ultimately the most important factor in determining its clinical utility. Lastly, and maybe the most important reason of all, 2D culture systems do not accurately replicate the complex microenvironment of a tumor, including interactions with stromal cells, immune cells, endothelial cells, and the extracellular matrix (ECM). In this context, it has already been shown that cell polarity, nuclear organization, and gene expression in tumor cells are affected by their interaction with the ECM^[13]^. In addition, tumor cells grown in three-dimensional (3D) culture systems exhibit clear differences in growth characteristics and response to chemotherapeutics compared to cells grown in conventional cell culture systems^[14-16]^. Over the past few years, significant progress has been made in developing more advanced culture systems that are able to more closely resemble the patient’s original tumor. For example, 3D tumor organoid cell culture models co-cultured with autologous immune cells or stromal cells have already been demonstrated to be a useful tool for studying the interaction of tumor cells with the tumor microenvironment (TME)^[17,18]^. Moreover, organ-on-chip technology has emerged as an innovative approach that integrates multiple cell types and can simulate the cellular and biochemical processes occurring in the TME^[19]^. Furthermore, 3D organotypic co-culture models have proven effective in maintaining the architecture and cell composition of the original tumor^[20]^. Interestingly, a 3D collagen-based scaffold model has been demonstrated to be a valuable tool for studying the TME and therapeutic resistance mechanisms in HNSCC^[21]^. Despite these advancements, it is important to acknowledge that current cell culture systems still have limitations in fully capturing the complexity of tumors and their interactions with the TME. As such, *in vivo* evaluation remains a crucial step in the drug development process and is necessary to ensure the safety and efficacy of novel treatment combination strategies, including cetuximab, before they can be approved for clinical use. However, to date, the availability of adequate *in vivo* mouse models specifically designed to study cetuximab resistance and sensitivity remains limited.

Historically, the working mechanism of cetuximab has largely been attributed to the direct effects of EGFR inhibition. However, cetuximab is also involved in processes that stimulate the immune system^[22-24]^. In this regard, cetuximab, being a chimeric human:mouse immunoglobulin G1 (IgG1), is able to mediate cellular immunity by inducing antibody-dependent cellular cytotoxicity (ADCC)^[25,26]^. This is a biological process where the fragment crystallizable (Fc) region of the antibody can bind to CD16 Fc receptors located on natural killer (NK) cells, macrophages and granulocytes, with NK cells being the most potent effectors^[27]^. This Fc-CD16 binding triggers the release of cytolytic proteins such as granzymes and perforin, leading to targeted destruction of tumor cells through apoptosis or lysis^[23,28]^. Moreover, studies have demonstrated that cetuximab has the ability to promote cross-priming of cytotoxic T cells via antigen-presenting cells such as dendritic cells^[29]^. This effect is primarily attributed to the induction of immunogenic cell death by cetuximab in tumor cells^[30]^. As such, the immune-mediated effects of cetuximab play a significant role in its antitumor activity. Therefore, in this study, we aimed to optimize two mouse models, with different cetuximab resistance status and intact ADCC functionality, that are able to subcutaneously grow tumors from human cell lines proven to be cetuximab-sensitive and -resistant *in vitro* and *in vivo*. Preservation of ADCC functionality in these mouse models is crucial to ensure that cetuximab can still execute not only EGFR inhibition but also mediate ADCC as part of its antitumor effects. Although partially immunodeficient, these mouse models are more representative of the human situation, since they are capable of executing ADCC, potentially improving the translatability of cetuximab responses from mice to humans. These two mouse models can be used in the future to test the potency of novel combination strategies containing cetuximab with the goal of overcoming resistance to cetuximab and exploring cetuximab resistance mechanisms in an *in vivo* setting.

## METHODS

### Cell lines and cell culture

We included three sets of isogenic cetuximab-sensitive versus acquired -resistant HNSCC cell lines. The SC263 cell line was kindly provided by Prof. Dr. Sandra Nuyts (University Hospital Leuven, Leuven, Belgium), the SCC22b cell line was kindly provided by Prof. Dr. Olivier De Wever (Laboratory of Experimental Cancer Research, Ghent University Hospital, Ghent, Belgium) and the FaDu-S and FaDu-R cell lines were kindly provided by Prof. Dr. Cyril Corbet (Pole of Pharmacology and Therapeutics, Institut de Recherche Expérimentale et Clinique, UCLouvain, Brussels, Belgium). Acquired-resistant variants (suffix R) of the initially cetuximab-sensitive SC263 and SCC22b cell lines were generated by chronic exposure to cetuximab as described previously by us^[7]^. In parallel, parental cell lines were exposed to the vehicle control, i.e., phosphate-buffered saline (PBS), and used as a control for vehicle exposure and an increased culture period (suffix S). Before inoculation, acquired-resistant cell lines were exposed to a high dose of cetuximab for 7 days to ensure proper selection of resistant cells. All cell lines were human papilloma virus (HPV)-negative and cultured in Dulbecco’s modified Eagle’s medium (DMEM, Gibco^TM^, 10938025), supplemented with 10% fetal bovine serum (FBS, Gibco^TM^, 10270106), 1% penicillin/streptomycin (Gibco^TM^, 15140122), and 2 mM L-glutamine (Gibco^TM^, 25030024). Cells were grown as monolayers and maintained in exponential growth in 5% CO_2_/95% air in a humidified incubator at 37 °C. Cell lines were confirmed free of mycoplasma infection through regular testing using the MycoAlert Mycoplasma Detection Kit (Lonza, LT07-118). The identity of each cell line was validated through short tandem repeat profiling.

### Animal facilities and animals

All animal care and testing were approved by the Ethics Committee of the University of Antwerp (N° 2020-41 and 2021-39) and performed according to the European guidelines within the facilities of the University of Antwerp, Campus Drie Eiken.

Female C57BL/6NRj-Rag2tm1Ciphe/Rj [Rag2 knock-out (KO)] mice, aged 4-6 weeks, were obtained from Janvier Labs. Female BALB/cAnN.Cg-Foxn1nu/Crl (BALB/c nude) and CB17/lcr-PrkdcScid/lcrlcoCrl (CB17 Scid) mice, aged 4-6 weeks, were obtained from Charles River Laboratories (supplier of The Jackson Laboratory in Europe). After arrival, mice were allowed to acclimatize for at least 7 days before being used in experiments to reduce stress levels. All mice were housed in controlled, specific pathogen-free environments with 12-hour cycles of light and dark and provided with food and clean water ad libitum. Mice were monitored daily for humane endpoints (body weight, appearance, behavior, and comorbidities). The number of mice varied through the experiments, taking into account both feasibility and ethical considerations. Adjustments were made based on the outcome of previous experiments, ensuring meaningful conclusions while minimizing the use of animals. The principle of reduction, refinement, and replacement (3Rs) was followed, leading to the use of minimal numbers in all experiments.

### Tumor kinetics and survival

**Figure 1:**
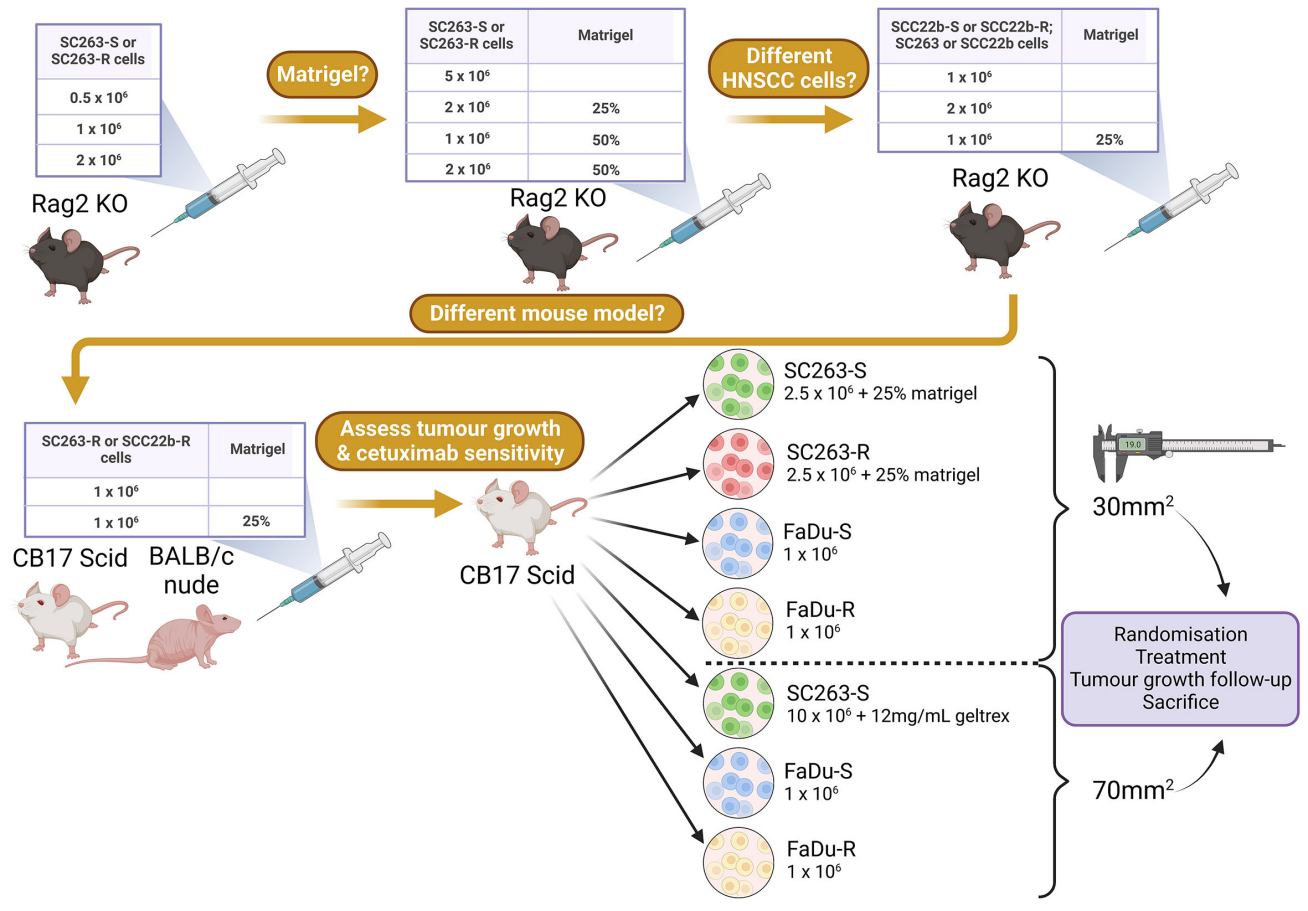
Schematic overview of performed experiments. This figure was created with Biorender.com. HNSCC: Head and neck squamous cell carcinoma; KO: knock-out; -R: cetuximab-resistant HNSCC cell line; -S: cetuximab-sensitive HNSCC cell line.

Prior to injection, tumor cells were harvested using TrypLE (Gibco^TM^, 12604021) and washed 3 times with sterile Dulbecco’s phosphate-buffered saline (DPBS, Gibco^TM^, 14190144). In all experiments, tumor cells were suspended in 100 µL sterile PBS and injected into the shaved hind flank of the mice. In each experiment, mice were injected subcutaneously with cetuximab-sensitive or -resistant HNSCC cells at different concentrations and with/without Matrigel or Geltrex according to the schematic overview in Figure 1. When tumors reached a size of approximately 30 or 70 mm^2^, mice were randomized based on tumor size and divided into different treatment groups. Tumor growth was monitored over time and measured two times a week using a digital caliper. Tumor size was calculated using the formula “length × width”. Mice were sacrificed when a tumor size of 150 mm^2^ was reached or when a humane endpoint was reached [Figure 1].

### In vivo administration of cetuximab

Mice were treated twice per week (with an interval of 3 to 4 days) with a low (2.5 mg/kg), medium (10 mg/kg), or high (50 mg/kg) dose of the anti-EGFR-targeted mAb cetuximab (Merck) or PBS control through intraperitoneal (i.p.) injection at the contralateral abdominal side of the tumor for a total treatment duration of 3 weeks. The doses (2.5, 10, and 50 mg/kg) were determined based on literature^[31-34]^. Iida *et al*. reported that no discernible toxicity was observed in mice treated with 50 mg/kg cetuximab twice a week for 10 consecutive weeks^[33]^. Calculations of the required cetuximab concentration in mg/kg were made for each individual mouse based on the individual body weight.

### Immunohistochemical analysis

SC263-R, FaDu-S, and FaDu-R tumors were harvested directly after the last week of cetuximab treatment (or before tumors disappeared completely), whereas tumors originating from the SC263-S cell line were collected without prior treatment, since inducing a durable tumor growth was challenging with this cell line. Formalin-fixed paraffin-embedded (FFPE, 5 µm thick) sections were prepared from tumor tissue blocks. Sections were incubated in a low pH buffer (pH6) for 20 min at 97 °C (PT-Link, DAKO) for heat-induced antigen retrieval. Peroxidase blocking buffer (3.5%, Acros Organics, 202465000) was used for 10 min to quench the endogenous peroxidase activity of the sections, followed by blocking with normal goat serum (for anti-F4/80, anti-Ki67 and cleaved caspase-3) or normal horse serum (for anti-NKp46). Subsequently, sections were incubated with primary antibodies: anti-NKp46 [1:50 for 60 min, NK cell marker, Bio-Techne (R&D Systems), polyclonal, AF2225-SP], anti-F4/80 (1:500 for 40 min, macrophage marker, Thermo Fisher Scientific, clone BM8, 14-4801-81), anti-Ki67 (1:400 for 35 min, proliferation marker, Cell Signaling Technology, clone D3B5, 12202S), and anti-cleaved caspase-3 (1:250 for 35 min, apoptosis marker, Cell Signaling Technology, polyclonal, 9661S). The ImmPRESS^TM^ goat anti-rabbit peroxidase kit (for anti-Ki67 and cleaved caspase-3, Vector, MP-7451), the ImmPRESS^TM^ goat anti-rat peroxidase kit (for anti-F4/80, Vector, MP-7444), or the ImmPRESS^TM^ horse anti-goat peroxidase kit (for anti-NKp46, Vector, MP-7405) in combination with the liquid DAB+ substrate chromogen system (DAKO, K3467) were used for signal detection according to the manufacturer’s instructions. All sections were counterstained with hematoxylin (0.1%, Merck, C.I.75290), dehydrated in a series of isopropanol baths (distilled water, 70%, 95%, 100%, Acros Organics, P/7490/FP21), cleared with xylene (MLS, ZY10020) and mounted with ExPert mounting medium (MLS, QC50082). Positive controls were included for each marker and consisted of mouse tissue of spleen (for anti-Ki67, anti-F4/80, and anti-NKp46) and lymph node (for anti-cleaved caspase-3). Pictures were taken with a Leica ICC50 E camera on a Leica DM500 microscope.

### Statistical analysis

Possible significant differences in tumor kinetics between treatment groups (*P* < 0.05) were evaluated with a linear mixed model by each time point with the treatment group as a fixed effect and the subject as a random effect using JMP Pro v16.0.0 software.

## RESULTS

### Rag2 KO mice prove to be ineffective as xenograft models for HNSCC tumor growth

Rag2 KO mice were selected as a suitable model for our first experiment, since this mouse strain has no mature B and T cells and is considered an excellent xenograft host for cancer cell lines. Importantly, the innate immunity is still intact in these mice. Thus, this model is highly suitable for testing the efficacy of therapeutic antibodies, such as cetuximab, as ADCC is still intact.

**Figure 2:**
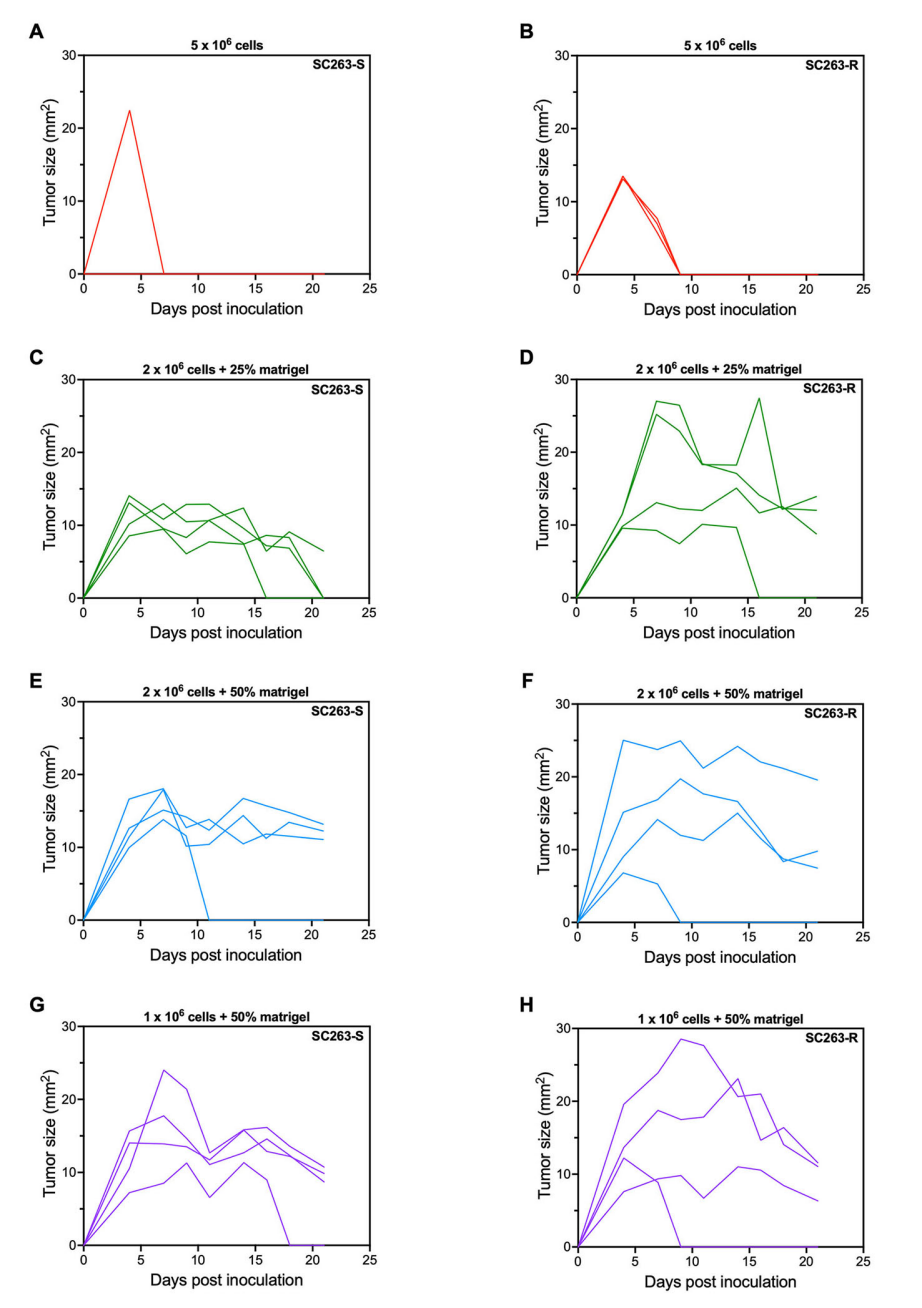
Co-injection of SC263 tumor cells with Matrigel in Rag2 KO mice did not result in sustained tumor growth. (A and B) Tumor kinetics after s.c. injection with 5 × 10^6^ SC263-S: (A) and SC263-R cells; (B) (*n* = 3); (C and D) Tumor kinetics after s.c. injection with 2 × 10^6^ SC263-S: (C) and SC263-R cells; (D) mixed with 25% Matrigel (*n* = 4); (E and F) Tumor kinetics after s.c. injection with 2 × 10^6^ SC263-S: (E) and SC263-R cells; (F) mixed with 50% Matrigel (*n* = 4); (G and H) Tumor kinetics after s.c. injection with 1 × 10^6^ SC263-S: (G) and SC263-R cells; (H) mixed with 50% Matrigel (*n* = 4). Each line represents the data of one individual mouse. KO: Knock-out; -R: cetuximab-resistant HNSCC cell line; -S: cetuximab-sensitive HNSCC cell line; s.c.: subcutaneous.

Rag2 KO mice were injected subcutaneously with a low (0.5 × 10^6^), medium (1 × 10^6^), and high (2 × 10^6^) number of the cetuximab-sensitive SC263-S and acquired cetuximab-resistant SC263-R cells and tumor growth was followed up over time. The SC263-S and SC263-R cells were selected, as these cell lines showed the most promising responses in *in vitro* combination experiments. Unfortunately, after 4 weeks of follow-up, no mouse had developed a measurable subcutaneous (s.c.) tumor. Therefore, we tried to achieve tumor growth by varying several conditions. Firstly, we co-injected the tumor cells with Matrigel (25% and 50%) since, according to literature, Matrigel co-injection can increase the initiation and growth of tumor cells *in vivo*^[35]^. In addition, a group of mice inoculated with 5 × 10^6^ cells (without Matrigel) was included, as some HNSCC xenograft studies using such high cell numbers have been described in literature^[36-38]^. After 2 weeks of follow-up, only the Matrigel groups showed measurable s.c. tumors that persisted for multiple measurements (≥ 3, Figure 2). However, some tumors completely disappeared over time, while most tumors of both cell lines slowly regressed, reaching a plateau without further exponential growth, and tumor sizes never reached 30 mm^2^ [Figure 2]. As a result, we decided to terminate this experiment, as it became evident that none of the groups would be appropriate for conducting further experiments.

**Figure 3:**
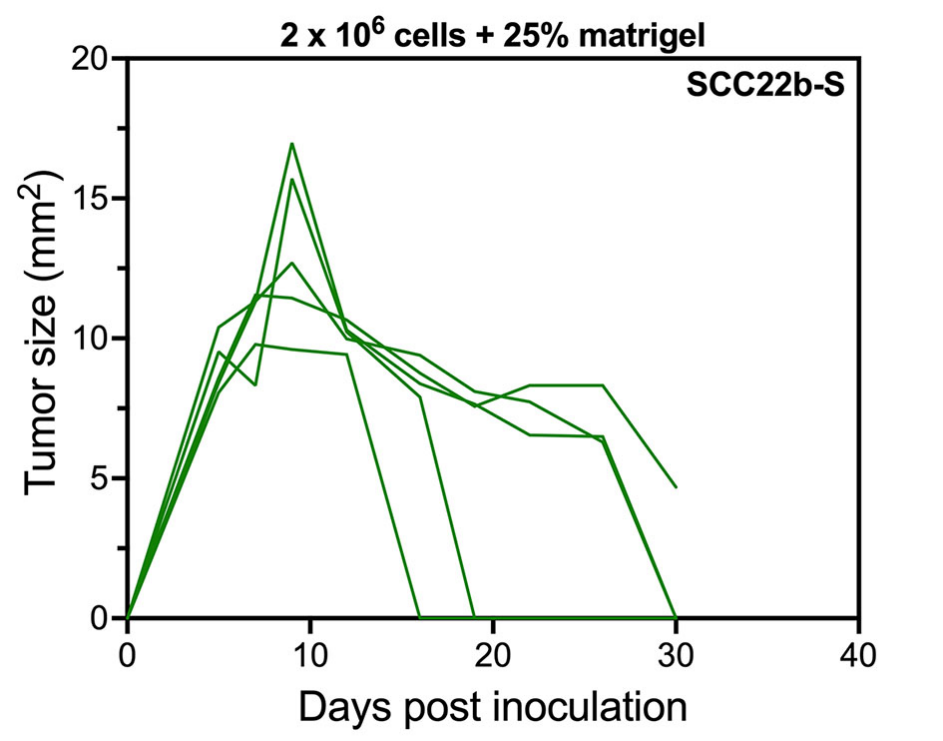
Tumor kinetics of SCC22b-S HNSCC cells with 25% Matrigel in a Rag2 KO mouse model. Tumor kinetics after s.c. injection with 2 × 10^6^ SCC22b-S mixed with 25% Matrigel (*n* = 5). Each line represents the data of one individual mouse. HNSCC: Head and neck squamous cell carcinoma; KO: knock-out; -S: cetuximab-sensitive HNSCC cell line; s.c.: subcutaneous.

Considering that the in-house developed SC263-S and SC263-R cell lines, as well as the parental SC263 cell line, have not been previously used in xenograft models to our knowledge, the results we obtained suggest that these cell lines may not be capable of initiating tumor growth *in vivo*. To further explore this hypothesis, we repeated the previous experiment with the SCC22b-S and SCC22b-R cell lines, with/without co-injection with Matrigel, as the parental SCC22b cell line has been employed in HNSCC xenograft studies before^[39-42]^, yet in other mouse models than Rag2 KO. Unfortunately, neither experimental group exhibited any sustainable tumor growth, indicating that these cell lines were also unsuitable as progressive HNSCC models in the Rag2 KO mouse strain. Although tumor growth was initially observed in mice injected with 2 × 10^6^ SCC22b-S cells with 25% Matrigel, this was very limited (tumor sizes < 17 mm^2^) and tumor size decreased rapidly over time [Figure 3]. To eliminate the possibility that the increased passage numbers of these cell lines due to in-house development of acquired resistance were causing the issue, we tested the parental SC263 and SCC22b cell lines in the Rag2 KO mouse strain with/without Matrigel, but without any success. In conclusion, we attempted several methods, including co-injection with Matrigel, adjusting cell number, multiple cell lines and testing parental cell lines, but none of them proved effective. These results led to the conclusion that the Rag2 KO mouse strain is unsuitable for xenografting HNSCC cell lines, at least for our sets of isogenic cetuximab-sensitive versus acquired -resistant cell lines.

### CB17 Scid mice prove to be a suitable host for resistant SC263-R HNSCC cells

In a subsequent pilot experiment guided by literature and advice from Janvier Labs, we evaluated the suitability of two different immunodeficient mouse models for xenografting human HNSCC cancer cell lines. More specifically, we injected BALB/c nude and CB17 Scid mice with SC263-R cells, both with and without 25% Matrigel, at a concentration of 1 × 10^6^ cells per injection. CB17 Scid mice are genetically engineered to have no T and B cells, but still have intact innate immunity, including NK cells. The BALB/c nude mice lack T cells, but not B cells. In addition, NK cells are present at normal levels, and therefore, this model is also suitable to investigate NK cell cytotoxic responses such as ADCC. For this pilot experiment, we chose to only evaluate the resistant variants of our cell lines.

**Figure 4:**
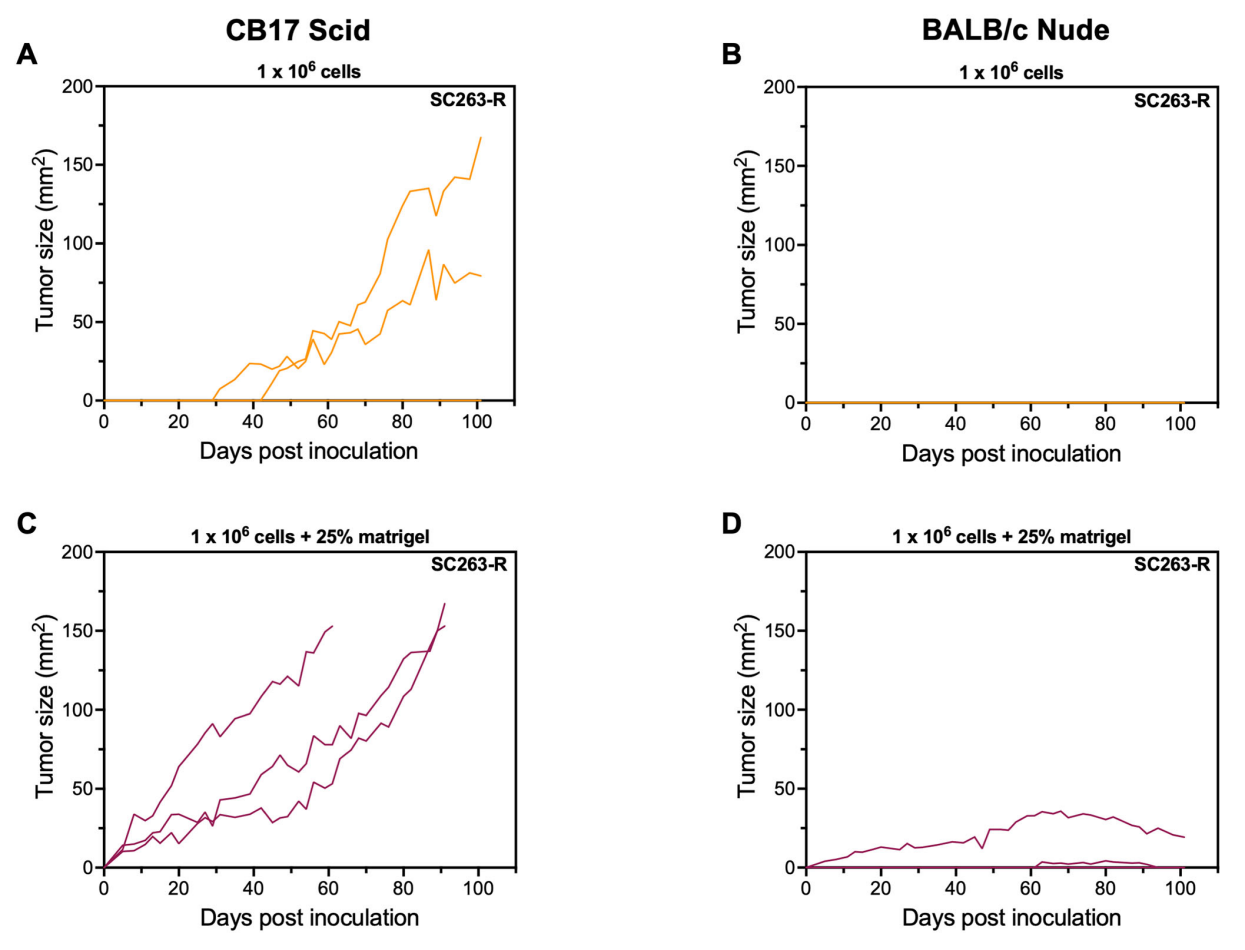
CB17 Scid mice are suitable hosts for SC263-R HNSCC cells when co-injected with 25% Matrigel. (A and B) Tumor kinetics over time after s.c. injection with 1 × 10^6^ SC263-R cells in CB17 Scid: (A) and BALB/c nude; (B) mice (*n* = 3); (C and D) Tumor kinetics over time after s.c. injection with 1 × 10^6^ SC263-R cells mixed with 25% Matrigel in CB17 Scid: (C) and BALB/c nude; (D) mice (*n* = 3). Each line represents the data of one individual mouse. HNSCC: Head and neck squamous cell carcinoma; -R: cetuximab-resistant HNSCC cell line; -S: cetuximab-sensitive HNSCC cell line; s.c.: subcutaneous.

Only two out of three CB17 Scid mice injected with 1 × 10^6^ SC263-R cells developed tumors, although very late in the experimental period, i.e., on days 31 and 45 post inoculation [Figure 4A]. In contrast, none of the BALB/c nude mice injected with only tumor cells showed any tumor growth [Figure 4B]. Interestingly, all CB17 Scid mice injected with 1 × 10^6^ tumor cells mixed with 25% Matrigel exhibited sustainable tumor growth, which was already measurable as early as day 5 post inoculation. These mice reached their endpoint (tumor size = 150 mm^2^) on days 61 and 91 [Figure 4C]. While two out of three BALB/c nude mice injected with tumor cells mixed with 25% Matrigel also developed tumors, growth was not sustained in this mouse strain [Figure 4D]. To maximize the information obtained from our pilot experiment and gain insight into the ability of SCC22b-R cells to grow in different mouse strains, mice that did not exhibit any tumor growth after injection with SC263-R cells were subsequently injected with SCC22b-R cells with Matrigel (for CB17 Scid mice) and with/without Matrigel (for BALB/c nude mice) at the opposite flank. However, none of the mice demonstrated sustainable tumor growth over time (data not shown). In conclusion, our pilot experiment suggested that the CB17 Scid mouse strain is an appropriate model for xenografting the human HNSCC SC263-R cell line in combination with Matrigel co-injection.

### Resistant SC263-R cells do not maintain their cetuximab resistance in CB17 Scid mice

**Figure 5:**
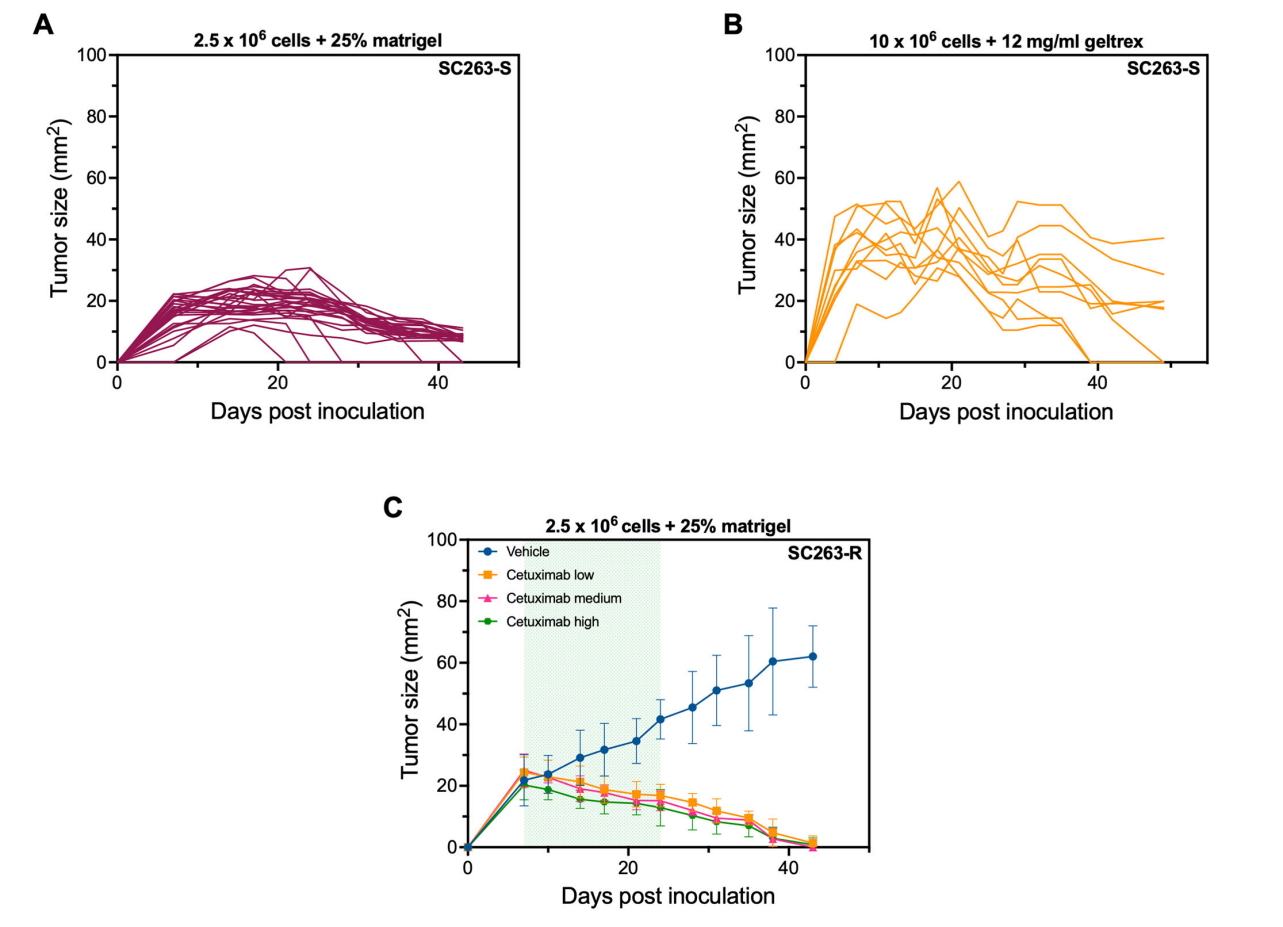
SC263 xenograft models in CB17 Scid mice are unsuitable for investigating cetuximab resistance. (A) Tumor kinetics after s.c. injection with 2.5 × 10^6^ + 25% Matrigel SC263-S cells in CB17 Scid mice (*n* = 27); (B) Tumor kinetics after s.c. injection with 10 × 10^6^ + 12 mg/mL Geltrex SC263-S cells in CB17 Scid mice (*n* = 10). Each line represents the data of one individual mouse; (C) Tumor kinetics of SC263-R tumor-bearing CB17 Scid mice following treatment with vehicle (PBS, *n* = 6), cetuximab low (2.5 mg/kg, *n* = 7), cetuximab medium (10 mg/kg, *n* = 7), and cetuximab high (50 mg/kg, *n* = 7). The green area in the graph represents the treatment period (starting from day 7). Data represent mean ± SD. HNSCC: Head and neck squamous cell carcinoma; -S: cetuximab-sensitive HNSCC cell line; -R: cetuximab-resistant HNSCC cell line; s.c.: subcutaneous.

Now that we have identified an appropriate mouse model, we proceeded to the next phase of our study, which involved testing the effectiveness of cetuximab. Dose titration of the EGFR-targeting mAb cetuximab was performed in order to investigate whether xenografted HNSCC cells retained their cetuximab sensitivity in an *in vivo* setting. As tumor growth was rather slow, we increased the number of HNSCC cells to be injected from 1 × 10^6^ to 2.5 × 10^6^ with 25% Matrigel. CB17 Scid mice were injected with either SC263-S or SC263-R cells to generate a model that is sensitive and resistant to cetuximab, respectively. When tumors reached a volume of approximately 30 mm^2^, mice were randomized into four treatment groups (vehicle, cetuximab low dose, cetuximab medium dose, and cetuximab high dose). Unfortunately, mice inoculated with the SC263-S cell line reached an average tumor size of a maximum of 25 mm^2^, after which the tumors spontaneously started to decrease in size for unknown reasons prior to treatment [Figure 5A]. In a final attempt to induce sustainable tumor growth of the SC263-S cell line in CB17 Scid mice, we remarkably increased the cell number and injected the mice with 10 × 10^6^ SC263-S cells. Due to a global shortage of Matrigel, we used 12 mg/mL Geltrex instead to promote tumor growth. Geltrex has successfully been used in our lab to grow solid tumors from hematological cancer cell lines and is therefore a good alternative for Matrigel. Unfortunately, the results were similar to the previous experiment using 2.5 × 10^6^ injected cells in CB17 Scid mice. Despite the tumors initially growing up to a maximum size of approximately 60 mm^2^, they eventually began to regress spontaneously [Figure 5B]. Hence, while the tumors in this experiment achieved a larger size than in the previous one, we were still unable to induce durable tumor growth.

In contrast, in the mice injected with the SC263-R cells, treatment could be initiated at day 7. Mice in the vehicle group showed linear tumor growth, whereas tumors in the treatment groups started to decrease from the moment of treatment initiation. This shrinkage of tumor volume in the treatment groups continued until the mice showed no visible/palpable tumor anymore [Figure 5C].

### Immunohistochemistry demonstrates the high presence of macrophages in SC263-R tumors induced in CB17 Scid mice

**Figure 6:**
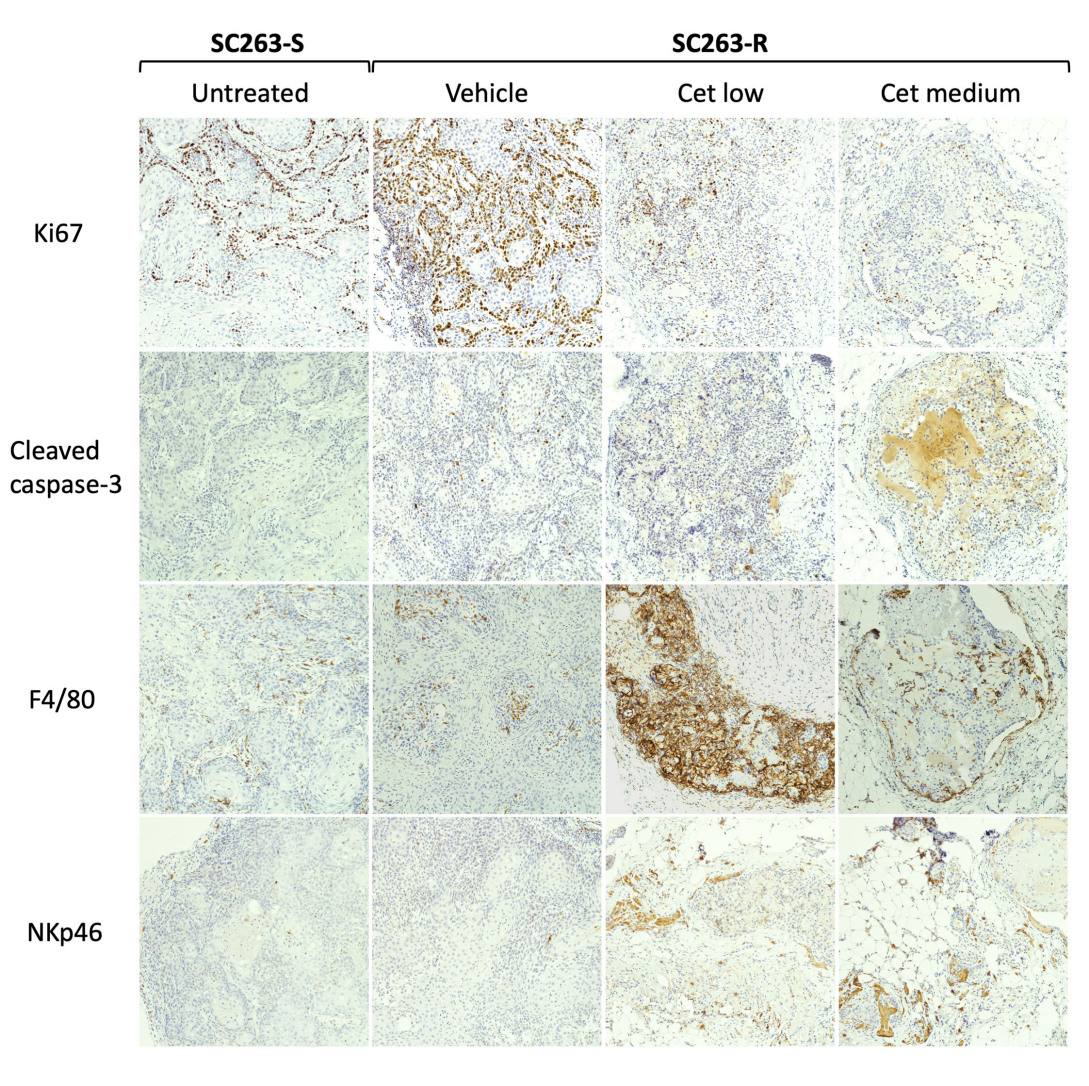
Infiltration of macrophages is likely compromising the growth of SC263-S tumors and the resistance to cetuximab of SC263-R tumors in CB17 Scid mice. Representative images of immunohistochemical staining for Ki67 (proliferation), cleaved caspase-3 (apoptosis), F4/80 (macrophages), and NKp46 (NK cells), shown at 100x. Vehicle: PBS; Cet low: 2.5 mg/kg cetuximab; Cet medium: 10 mg/kg cetuximab. NK: Natural killer; PBS: phosphate-buffered saline; -R: cetuximab-resistant HNSCC cell line; -S: cetuximab-sensitive HNSCC cell line.

As the CB17 Scid mouse strain has an intact innate immune system, the above-mentioned results might be explained by an activation of innate immune cells. To investigate this, we harvested tumors from both SC263-S and SC263-R tumor-bearing mice and performed immunohistochemistry [Figure 6 and Supplementary Figure 1]. Untreated SC263-S tumors were characterized by a low to moderate proliferation rate, little to no apoptotic cells, a moderate abundance of macrophages, and little to no NK cells. In contrast, Ki67 staining in vehicle-treated SC263-R-tumor-bearing mice indicated a high proliferation rate in these tumors. However, cetuximab treatment resulted in lower Ki67+ cells, indicating that cetuximab was inhibiting tumor cell proliferation in resistant cells. Although higher compared to SC263-S tumors, cleaved caspase-3 staining in SC263-R tumors was limited and slightly increased in the treatment groups. F4/80+ macrophages unexpectedly infiltrated the cetuximab-treated SC263-R tumors (particularly in the cetuximab low treatment group). This suggests that cetuximab may be altering the tumor microenvironment by increasing the infiltration of macrophages into the tumor, causing the *in vitro* resistant cells to lose their resistance *in vivo*. Little to no NKp46+ cells were present in the vehicle group, but when tumors were treated with cetuximab, more NK cells appeared. However, this increase was not as pronounced as the infiltration of macrophages observed in treatment groups. Overall, these results suggest that there is a predominant presence of macrophages in both SC263-S and SC263-R tumor-bearing mice, which may be impairing the growth of SC263-S cells and the resistance of SC263-R to cetuximab *in vivo*.

### FaDu cell lines retain their in vitro sensitivity status to cetuximab in CB17 Scid mice

**Figure 7:**
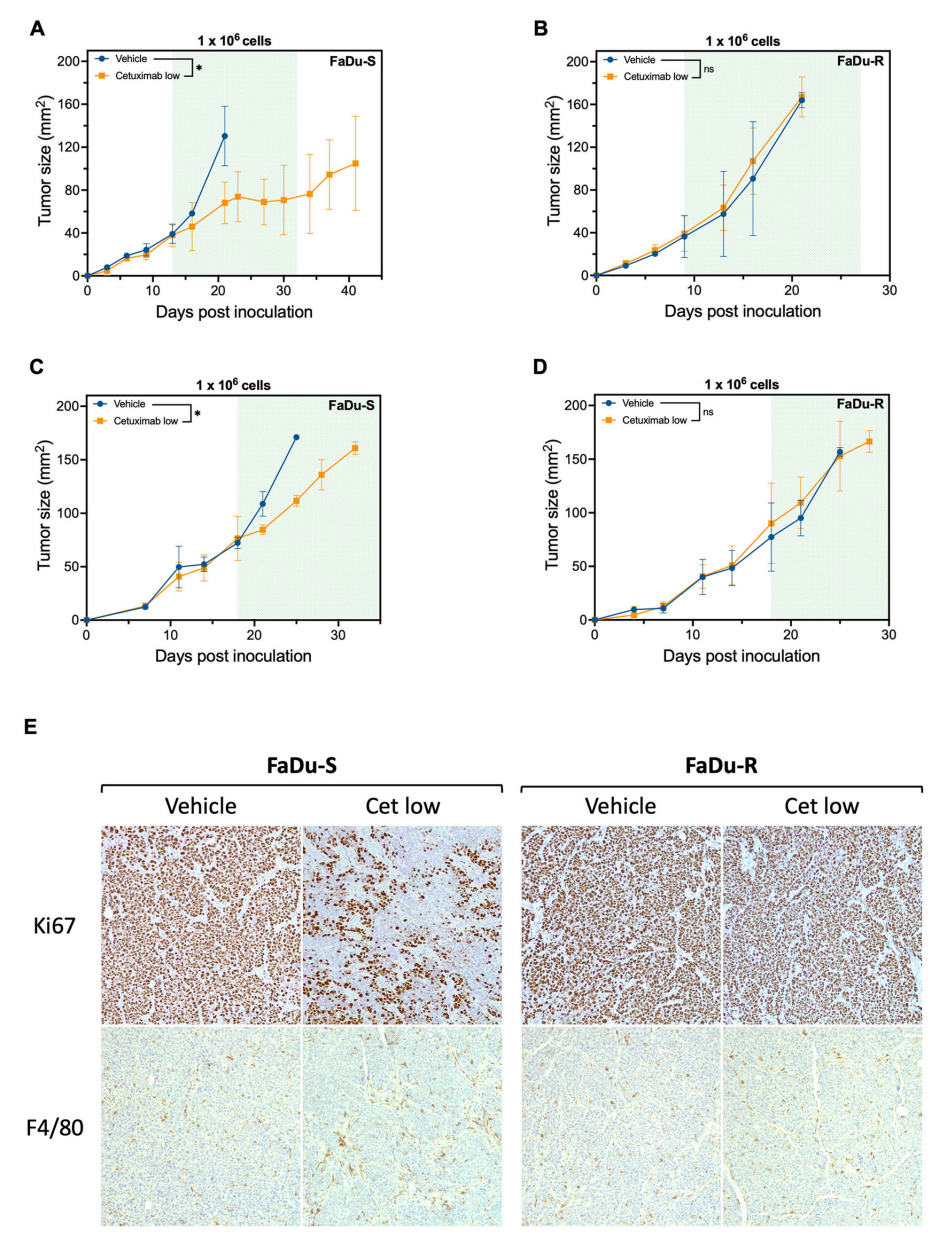
Cetuximab resistance status of isogenic FaDu cell lines is maintained in CB17 Scid mice. (A-D) Tumor kinetics of CB17 Scid mice inoculated with 1 × 10^6^ cetuximab sensitive FaDu-S cells (A and C) or cetuximab resistant FaDu-R cells (B and D) following treatment with vehicle (PBS, *n* = 2) or cetuximab low (2.5 mg/kg, *n* = 3). The green area in each graph represents the treatment period [starting from (A) day 13, (B) day 9 or (C and D) day 18]. Data represent mean ± SD. *P*-values were determined using a linear mixed model; (E) Representative images of immunohistochemical staining for Ki67 (proliferation) and F4/80 (macrophages), shown at 100x. *P < 0.05. ns: Non-significant; PBS: phosphate-buffered saline; -R: cetuximab-resistant HNSCC cell line; -S: cetuximab-sensitive HNSCC cell line.

In a final attempt to establish a reliable *in vivo* model for cetuximab resistance, we changed the HNSCC cell lines from SC263 to FaDu (sensitive and acquired resistant variant). In a pilot experiment with these cell lines in CB17 Scid mice, both the FaDu-S and FaDu-R cell lines exhibited robust tumor growth without the need to add Matrigel or Geltrex [Supplementary Figure 2A and B]. This stands in contrast to the SC263-S cell line, which failed to demonstrate sustainable tumor growth in this mouse model [Figure 5A and B]. To investigate whether FaDu cells maintained their *in vitro* resistance status to cetuximab, mice were injected with 1 × 10^6^ FaDu-S or FaDu-R cells. When tumors reached a size of approximately 30 mm^2^, mice were randomized and treated with vehicle or a low dose of cetuximab (2.5 mg/kg). We opted to test only a low dose of cetuximab, since tumors already completely disappeared with this dosage in SC263-R tumor-bearing CB17 Scid mice [Figure 5C]. Treatment was initiated on days 13 and 9 post-inoculation for mice with FaDu-S and FaDu-R tumors, respectively. Treatment with cetuximab resulted in a significant delay in tumor growth in FaDu-S-bearing mice, while FaDu-R-bearing mice demonstrated persistent tumor growth upon cetuximab treatment [Figure 7A and B, Supplementary Figure 2C and D].

Since tumor size has been reported to be inversely correlated to response to EGFR inhibitors and chemotherapy^[43-45]^ in patients and CBA/lac mice, we next investigated the response to cetuximab in more established tumors in both models by delaying treatment initiation to a tumor size of approximately 70 mm^2^. In these more established models, FaDu-S and FaDu-R tumor-bearing mice reached the treatment initiation point on day 18 post inoculation. Cetuximab treatment effectively reduced tumor growth exclusively in the FaDu-S tumor-bearing mice [Figure 7C and D], indicating that even in more established and advanced tumors, cetuximab resistance status is maintained. However, due to the study’s implemented humane endpoints, the treatment window was too small to complete the intended three-week treatment period. To obtain more insight into the tumor and its microenvironment, immunohistochemistry was performed and demonstrated a remarkable decrease in Ki67+ cells and a slight increase in macrophages after cetuximab treatment in these tumors [Figure 7E]. The former confirms that cetuximab effectively reduced tumor proliferation in cetuximab-sensitive FaDu-S tumors but not in resistant FaDu-R tumors. Cetuximab treatment had no effect on the level of apoptosis or NK cell infiltration [Supplementary Figure 2E]. In conclusion, the FaDu-S and FaDu-R cell lines effectively maintain their *in vitro* resistance status to cetuximab *in vivo* in CB17 Scid mice, even when tumors are more established. As such, these robust and reliable models underscore the suitability and promise of using xenografts with both sensitive and acquired resistant FaDu HNSCC cell lines for investigating novel combination therapies aimed at overcoming cetuximab resistance in an *in vivo* setting.

## DISCUSSION

Cetuximab resistance poses a significant challenge in the search for effective treatment options for HNSCC. To understand and address this problem, it is crucial to develop *in vivo* models that accurately depict cetuximab resistance. Such clinically relevant animal models are essential for studying and exploring novel combinations that have the potential to overcome resistance to cetuximab. Over the years, xenografts have become the gold standard for investigating novel cancer treatments, as they allow the use of human cell lines or even patient samples^[46]^. However, the majority of studies addressing novel cancer treatments in HNSCC have primarily relied on xenograft models using human cell lines, without specifically focusing on resistance nor on retaining intact ADCC functionality *in vivo*. Studies specifically using an acquired cetuximab-resistant xenograft model in HNSCC are rather scarce and appear to be challenging to develop^[47,48]^. As far as our knowledge extends, our research represents the first successful establishment of an acquired cetuximab-resistant HNSCC model derived from *in vitro*-generated acquired resistant cells. In addition to our work, there have been previous attempts to establish *in vivo* models of acquired cetuximab resistance by chronically treating tumor-bearing mice with increasing doses of cetuximab, however, without success for HNSCC^[47,48]^. There has also been a study that established an HNSCC *in vivo* mouse model by utilizing cells with the EGFR-K521 polymorphism that are intrinsically resistant to cetuximab^[49]^. However, our work specifically focuses on acquired resistance, making our study distinct and unique in the field. In contrast, more success has been achieved with patient-derived xenograft mouse models for acquired cetuximab resistance, offering a valuable alternative and clinically relevant approach to study acquired cetuximab resistance *in vivo*^[50,51]^.

In the present study, we aimed to establish a xenograft model of acquired resistance using a human HNSCC cell line that was initially made resistant *in vitro* by chronically exposing it to increasing doses of cetuximab^[7]^. Our attempts to generate a resistance model using the Rag2 KO mouse strain were unsuccessful, despite various attempts to optimize tumor growth induction by adjusting tumor cell number, cell lines and/or the addition of Matrigel. The finding that not even the SCC22b-S, which has only a larger passage number than its parental cell line, failed to induce sustainable tumor growth is particularly surprising, considering the extensive use of the parental cell line in numerous published xenograft studies^[39-42]^, yet in other mouse models than Rag2 KO and not focusing on cetuximab resistance. Initially, we chose the Rag2 KO mouse model, since this strain has been reported as a suitable host for tumor implantation studies of several cancers, including HNSCC^[52-55]^. This is because the loss of Rag2 blocks the development of mature B and T cells, creating an immunodeficient mouse model that should be able to accept allogenic transplants^[55-57]^. The C57BL/6N genetic background of the mouse strain employed in our study could potentially explain the unsuccessful tumor engraftment. C57BL/6N mice have a more robust and aggressive innate immunity in comparison to mice with a BALB/c background^[58-62]^. This more robust immune activity could have triggered an amplified immune response against the implanted tumor cells, ultimately inhibiting their growth and engraftment in the Rag2 KO mouse strain.

Next, we conducted a pilot experiment using CB17 Scid and BALB/c Nude mice, two different mouse strains on a BALB/c genetic background with intact innate immunity that have been extensively used in HNSCC xenograft studies^[37,41,63-65]^, although not specifically in the context of cetuximab. Based on this experiment, the CB17 Scid mouse strain was selected as a suitable model for xenografting the SC263-R cell line. It is important to note that a limitation of this pilot experiment was the exclusion of the sensitive cell line (SC263-S), primarily due to the limited availability of mice, which was later found to be unable to grow in this specific mouse strain. Additionally, we observed that the acquired cetuximab-resistant variant SC263-R had lost its resistance phenotype in this specific *in vivo* model. This observation has been reported in literature before, although to a limited extent. In this regard, the study of Formelli *et al*. showed that doxorubicin-resistant B16 melanoma cells only maintained their resistance *in vivo* when the *in vitro* resistance index was greater than 100^[66]^. Similarly, when the *in vitro*-derived cetuximab-resistant SCC1c8 HNSCC cell line was transplanted into an athymic nude mouse model, it unexpectedly lost its resistance phenotype^[48]^. This mouse model lacks T cells but has functional B cells, NK cells and macrophages^[67]^, which may have been the reason for the observed loss of resistance *in vivo.* Similarly, an *in vitro* generated breast cancer cell line resistant to (ADCC-capable) trastuzumab failed to maintain its resistance status in an *in vivo* mouse model^[68]^. These outcomes highlight the limitations of solely relying on *in vitro* drug exposure to generate resistant clones. To ensure more reliable *in vivo* models of drug resistance, it is suggested that *in vivo* selection or a combination of *in vivo* and *in vitro* selection methods should be employed^[69]^. However, *in vivo* selection is still challenging and does not guarantee the successful generation of resistance models. This was demonstrated by Quesnelle *et al*., who performed an *in vivo* selection of 10 different HNSCC cell lines, including SCC22b, with the goal of establishing much-needed *in vivo* models of cetuximab resistance. However, despite their efforts, none of the cell lines exhibited successful acquisition of cetuximab resistance in their *in vivo* setting^[47]^. In addition, *in vivo* generated cetuximab-resistant cancer cells demonstrated to slowly lose their resistance phenotype after several *in vitro* passages^[70,71]^. The cetuximab-resistant cell lines used in the present study were solely generated *in vitro*, but have proven to maintain their resistance phenotype even after 6 weeks of culture without cetuximab^[7,72]^, excluding the latter as a possible reason for the loss of resistance *in vivo*.

As immunohistochemical analysis revealed a high presence of macrophages in both SC263-S and SC263-R tumors, these macrophages might have impaired the growth of SC263-S cells and the resistance phenotype of SC263-R cells. The presence of macrophages in the tumor microenvironment may have exerted suppressive effects on tumor growth by contributing to an antitumor immune response or by directly influencing tumor cell behavior. Nevertheless, we acknowledge that our immunohistochemical findings warrant further validation with larger sample sizes to ensure robustness and reliability before drawing any definitive conclusions. In addition, factors such as oxygen levels, nutrient availability, cell-cell interactions and the presence of stromal cells, which are all different or even absent in *in vitro* cell cultures, may influence the behavior of tumor cells and potentially impact their response to cetuximab. In this regard, it has been shown that hepatocellular cancer cells cultivated *in vitro* in more native conditions exhibited an altered drug sensitivity compared to cells cultured in standard conditions^[73]^, highlighting the influence of the tumor microenvironment on drug sensitivity^[74]^.

In a final attempt to establish a cetuximab-resistant *in vivo* model from an *in vitro* generated resistant HNSCC cell line, we utilized acquired resistant FaDu-R cells. The parental FaDu cell line has already been used in HNSCC xenograft studies^[64,75-77]^, and both FaDu-S and FaDu-R have been demonstrated to induce robust tumor growth in CB17 Scid mice. Moreover, we showed that both FaDu-S and FaDu-R cell lines maintain their sensitivity/resistance status to cetuximab *in vivo*, in contrast to our other *in vitro*-proven cetuximab-resistant HNSCC cell lines.

Previous studies in mice have demonstrated that more advanced tumors have a lower response to EGFR inhibitors and chemotherapy^[43,44]^. Together with the fact that larger gross tumor volumes have been linked to worse outcomes in HNSCC patients receiving cetuximab and radiotherapy^[73]^, we delayed treatment initiation until the tumor was more established, as it might lead to reduced responsiveness to cetuximab. Yet, FaDu-S and FaDu-R cells maintained their resistance phenotype when treatment was initiated at a larger tumor size. However, they exhibited rapid growth, failing to complete the intended three-week treatment period. Overall, this demonstrates that we have succeeded in establishing reliable and robust HNSCC mouse models, where the cetuximab resistance status of the tumor cells remains unaffected by larger tumor sizes.

It is important to mention that our models for cetuximab resistance have certain limitations. More specifically, we established tumor models using both cetuximab-sensitive and -resistant variants derived from only one HPV-negative cell line of hypopharyngeal origin, restricting our models to fully capture the heterogeneity and complexity of the HNSCC patient population. Indeed, one single HNSCC cell line may not fully capture the diverse molecular and phenotypic profiles observed in different patients. The choice to focus only on HPV-negative HNSCC can be justified by the fact that this patient population is in greater need of novel treatment options, as they have an inferior prognosis in terms of recurrence and survival compared to HPV-positive patients^[78]^. In addition, HPV-positive HNSCC patients are, in the majority of cases, intrinsically resistant to cetuximab^[79,80]^, making an acquired cetuximab-resistant *in vivo* model for this patient population less clinically relevant. It is also worth mentioning that our established models are only validated for low doses (2.5 mg/kg) of cetuximab with a treatment period of three weeks. Further validation of the models with higher doses is still required. Furthermore, since our models are xenograft models, these mice lack a fully functional immune system, specifically the adaptive immunity component, which plays a crucial role in the evaluation of immunotherapeutic agents, such as pembrolizumab and nivolumab^[81]^. However, previous studies in literature have utilized similar mouse strains with only ADCC induction capability and no adaptive immunity to evaluate cetuximab-containing treatment combinations^[31,32,40,82-84]^, highlighting these models as valuable research tools. Although a syngeneic model would address the limitation of lacking adaptive immunity in our CB17 Scid mouse models, its use was not considered suitable for our study due to the inability of cetuximab to bind to murine EGFR^[85]^. While there is a mouse variant of cetuximab known as 7A7, which was initially proposed as a valuable antibody for EGFR-based preclinical studies in mice^[86]^, a recent study failed to reproduce the earlier reported results^[87]^. Furthermore, apart from the laboratory that initially reported 7A7, there are no other published studies in literature that have used this specific antibody, despite the first report dating back 20 years. Considering these factors, we opted against using a syngeneic model for our study, as it would not have provided the necessary compatibility with cetuximab and an accurate representation of its effects in mice. Alternatively, humanized mouse models could be a more optimal choice, as they possess a complete human immune system, including human NK cells^[81]^. However, humanized mouse models can be costly to establish and maintain, making them a financial challenge for many research laboratories. Therefore, our models consider a robust and economical approach for cetuximab resistance and combination studies *in vivo*.

In conclusion, we have successfully established *in vivo* mouse models for cetuximab resistance and sensitivity using the FaDu-R and FaDu-S cell lines, respectively, in CB17 Scid mice with intact ADCC functionality. These models provide a useful tool for studying resistance mechanisms and novel drug combination strategies in a more clinically relevant setting.

## DECLARATIONS

### Availability of data and materials

All data generated or analyzed during this study are available from the corresponding author upon reasonable request.

### Copyright

© The Author(s) 2023.
