# Supplementary material for: Establishment of head and neck squamous cell carcinoma mouse models for cetuximab resistance and sensitivity
Source: Cancer Drug Resist. 2023 Oct 17;6(4):709–28. doi: 10.20517/cdr.2023.62 (PMC10792481; doi:10.20517/cdr.2023.62)
Supplement: Supplementary file 1 [file cdr-6-4-709-SupplementaryMaterials.pdf]

Zaryouh *et al. Cancer Drug Resist* 2023;6:xx
DOI: 10.20517/cdr.2023.62

Cancer
Drug Resistance

Supplementary Materials

Establishment of head and neck squamous cell carcinoma mouse models for cetuximab resistance and sensitivity

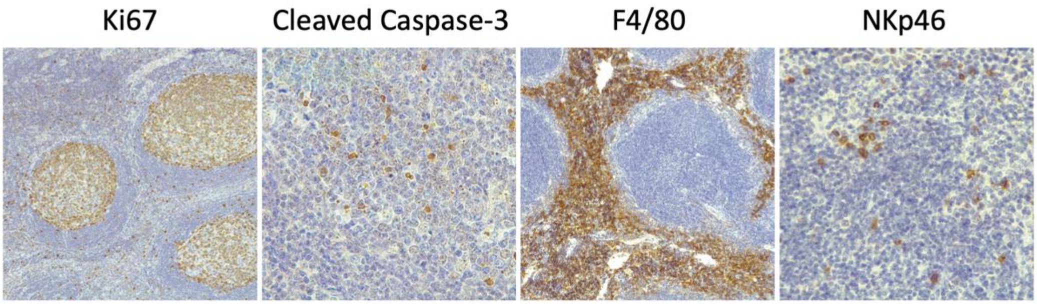

Supplementary Figure 1. Positive controls for immunohistochemistry. Positive controls for each marker consisted of mouse tissue of spleen for immunostaining with anti-Ki67 (shown at 100x), anti-F4/80 (shown at 100x) and anti-NKp46 antibodies (shown at 400x) and lymph node for detection of cleaved caspase-3 (shown at 400x).

© The Author(s) 2023. Open Access This article is licensed under a Creative Commons Attribution 4.0 International License (https://creativecommons.org/licenses/by/4.0/), which permits unrestricted use, sharing, adaptation, distribution and reproduction in any medium or format, for any purpose, even commercially, as long as you give appropriate credit to the original author(s) and the source, provide a link to the Creative Commons license, and indicate if changes were made.

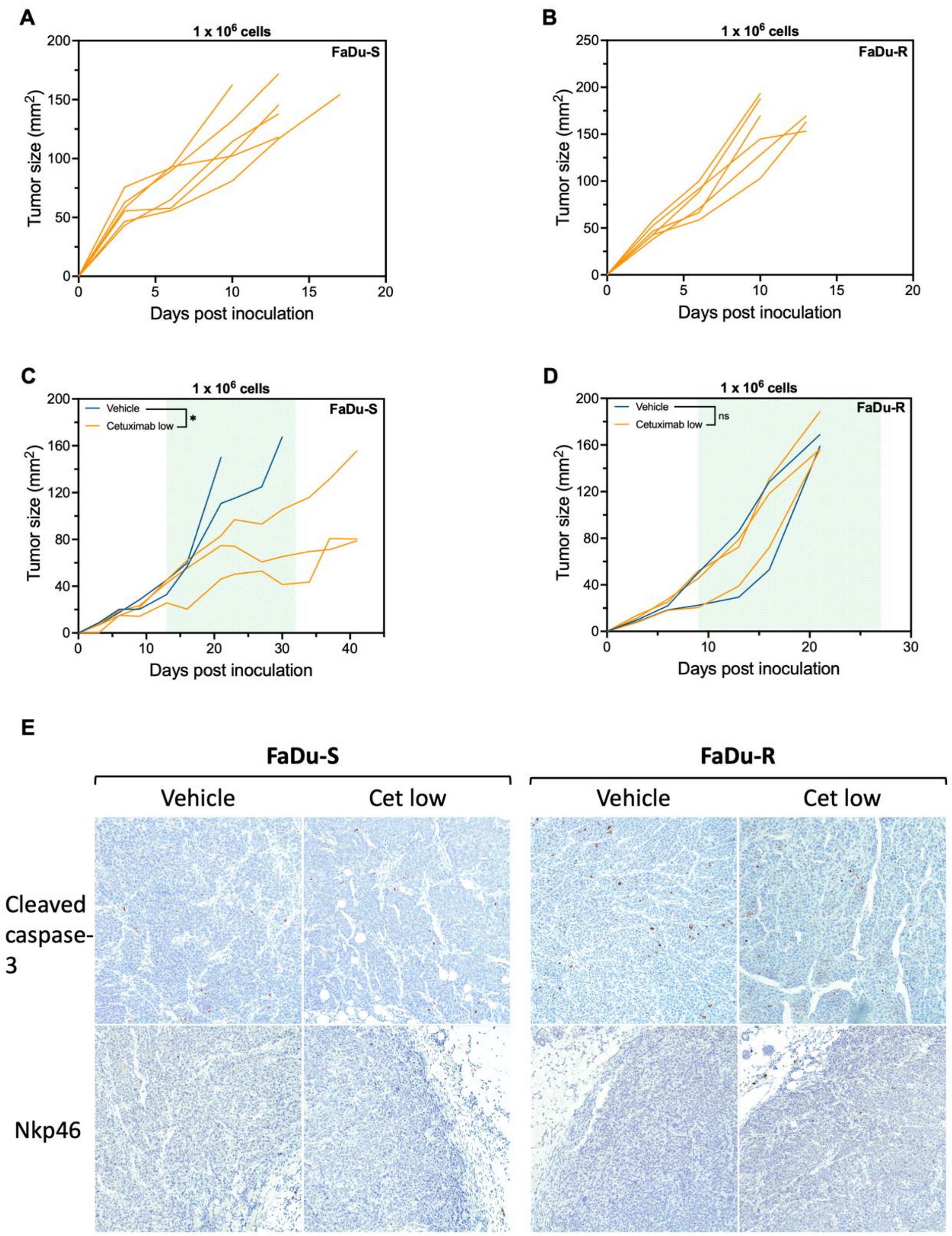

Supplementary Figure 2. Cetuximab had no effect on apoptosis or NK infiltration in FaDu HNSCC cell lines that retained their resistance status *in vivo*. (A-B) Tumor kinetics of CB17 Scid mice inoculated with 1 × 10^6 cetuximab-sensitive FaDu-S cells (A, n = 6) or cetuximab-resistant FaDu-R cells (B, n = 6); (C-D) Tumor kinetics of CB17 Scid mice after s.c. injection with 1 × 10^6 cetuximab-sensitive FaDu-S cells (C) or cetuximab-resistant FaDu-R cells (D) following treatment with vehicle (PBS, n = 2) or cetuximab low (2.5 mg/kg, n = 3). Treatment was initiated when tumors reached an

average size of 30 mm^2, which is represented by the green area in each graph. Each line represents the data of one individual mouse. *P*-values were determined using a linear mixed model; (E) Representative images of immunohistochemical staining with anti-cleaved caspase-3 anti-cleaved caspase-3 (apoptosis), and anti-NKp46 (NK cells) antibodies, shown at 100x. Vehicle: PBS; cet low: 2.5 mg/kg cetuximab; cet mid: 10 mg/kg cetuximab; $*P < 0.05$; ns: non-significant; -S: cetuximab sensitive cell line; -R: cetuximab resistant cell line.
